# Melatonin Ameliorates MI-Induced Cardiac Remodeling and Apoptosis through a JNK/p53-Dependent Mechanism in Diabetes Mellitus

**DOI:** 10.1155/2020/1535201

**Published:** 2020-01-09

**Authors:** Linhe Lu, Jipeng Ma, Mingming Sun, Xiaowu Wang, Erhe Gao, Lintao Lu, Jun Ren, Lifang Yang, Jian Yang

**Affiliations:** ^1^Department of Cardiovascular Surgery, Xijing Hospital, The Air Force Medical University, 710032, China; ^2^Center for Cardiovascular Research and Alternative Medicine, University of Wyoming, Laramie, WY 82071, USA; ^3^Center for Translational Medicine, Lewis Katz School of Medicine at Temple University, Philadelphia, PA 19140, USA; ^4^Department of Cardiology, Xijing Hospital, Air Force Medical University, Xi'an, China; ^5^Department of Anesthesiology, Xi'an Children's Hospital, Xi'an 710003, China

## Abstract

Diabetes mellitus, a worldwide health threat, is considered an independent risk factor for cardiovascular diseases. The overall cardiovascular risk of diabetes is similar to the one having one myocardial infarction (MI) attack although the precise impact of diabetes on MI-induced myocardial anomalies remains elusive. Given that mortality following MI is much greater in diabetic patients compared to nondiabetic patients, this study was designed to examine the effect of melatonin on MI injury-induced myocardial dysfunction in diabetes. Adult mice were made diabetic using high-fat feeding and streptozotocin (100 mg/kg body weight) prior to MI and were treated with melatonin (50 mg/kg/d, p.o.) for 4 weeks prior to assessment of cardiac geometry and function. The MI procedure in diabetes displayed overt changes in cardiac geometry (chamber dilation and interstitial fibrosis) and functional anomalies (reduced fractional shortening and cardiomyocyte contractile capacity) in association with elevated c-Jun N-terminal kinase (JNK) phosphorylation and p53 level. Melatonin treatment markedly attenuated cardiac dysfunction and myocardial fibrosis in post-MI diabetic mice. Furthermore, melatonin decreased JNK phosphorylation, reduced p53 levels, and suppressed apoptosis in hearts from the post-MI diabetic group. *In vitro* findings revealed that melatonin effectively counteracted high-glucose/high fat-hypoxia-induced cardiomyocyte apoptosis and contractile dysfunction through a JNK-mediated mechanism, the effects of which were impaired by the JNK activator anisomycin. In summary, our study suggests that melatonin protects against myocardial injury in post-MI mice with diabetes, which offers a new therapeutic strategy for the management of MI-induced cardiac injury in diabetes.

## 1. Introduction

Type 2 diabetes mellitus (T2DM) is a major public health threat worldwide and triggers severe clinical complications such as diabetic cardiomyopathy, retinopathy, nephropathy, and neuropathy [1–4]. Importantly, it is well recognized that T2DM is an independent risk factor for coronary heart diseases [5]. Ample clinical studies have provided compelling evidence that diabetic patients suffer from an unfavorable prognosis following myocardial infarction (MI) [6]. In particular, the 28-day mortality after MI nearly doubles in diabetic patients compared with nondiabetic patients [7]. However, despite its clinical importance, the impact of MI on myocardial geometry and function remains somewhat obscure in diabetes. Thus, it is pertinent to elucidating the underlying molecular mechanisms behind MI-induced cardiac geometric and contractile anomalies in diabetes mellitus, in an effort to explore novel and better therapeutic options against this devastating comorbidity.

Melatonin is a hormone secreted from the pineal gland found in nearly all organisms. In addition to its well-known roles in the circadian rhythm of sleep and antioxidant regulation [8, 9], melatonin was reported to preserve liver function from streptozotocin-induced diabetes [10–12] as well as to alleviate left ventricular remodeling and cardiac dysfunction after MI through apoptosis inhibition [13–15]. Importantly, it was shown that the low level of nocturnal serum melatonin was associated with not only acute myocardial infarction but also left ventricular remodeling in patients following acute MI [16, 17]. Moreover, recent evidence suggested that melatonin dramatically attenuated post-MI injury through regulating the Notch1/Mfn2 pathway and reducing ROS generation [18–20]. Nonetheless, the possible impact of melatonin on post-MI-induced cardiac injury has not been carefully delineated in diabetes. To this end, the effect of melatonin on post-MI-induced cardiac anomalies was examined in diabetes.

Activation of the mitogen-activated protein kinase (MAPK) stress signaling has been well documented in both ischemic and diabetic heart diseases [21, 22]. MAPKs mainly are composed of three well-defined protein kinases including the extracellular signal-regulated kinases (ERKs), the c-Jun NH_2_-terminal kinases (JNKs), and the p38 enzymes (p38 MAPKs), to regulate a wide array of cellular activities including mitosis, metabolism, and programmed cell death [23]. Importantly, inhibition of JNK using the JNK inhibitor SP600125 or endogenous macrophage migration inhibitory factor significantly reduced cardiac ischemia-reperfusion injury [24, 25]. Furthermore, doxorubicin-induced JNK activation provoked cardiac apoptosis and functional abnormalities [26]. A novel curcumin derivative, namely, C66, was found to attenuate diabetic cardiomyopathy through inhibition of JNK phosphorylation [27, 28]. Although JNK serves as a key player in multiple pathological settings of the heart, the function of JNK in post-MI injury with diabetes needs further elucidation.

## 2. Materials and Methods

### 2.1. Animals and Experimental Protocol

This study was performed according to the National Institutes of Health Guidelines on the Use of Laboratory Animals (National Institutes of Health Publication No. 8523, revised 1996), and experimental protocol herein was approved by the Air Force Medical University Institutional Committee on Animal Care. In brief, male C57BL/6J mice (18-22 g) at the age of 8-10 weeks were purchased from the Experimental Animal Center of the Air Force Medical University. Mice were housed with access to normal diet and water *ad libitum* at 23-25°C and were acclimatized for 1 week under a 12 hr/12 hr light/dark cycle. Mice were then randomly divided into the following groups: (I) The normal control group (CON) was fed standard chow *ad libitum* for 4 weeks. Mice were fasted overnight before they were injected intraperitoneally with an equal volume (100 *μ*L) of 0.1 M citrate buffer for two consecutive days. Mice were raised for another 6 weeks after sham operation. (II) The type 2 diabetes group (T2DM) was fed high-fat diet (D12451, 45% kcal from fat, Research Diets, New Brunswick, NJ, USA) for 4 weeks prior to overnight fasting. Mice were then given 100 *μ*L solution of streptozotocin (S0130, Sigma-Aldrich, MO, USA, 50 mg/kg body weight/d, dissolved in 0.1 M citrate buffer, pH 4.5, i.p.) for two consecutive days. Mice were deemed diabetic with fasting plasma glucose (FPG) of >11.1 mmol/L 3 days after injection. After that, mice were randomly assigned into the following three groups: (IIa) type 2 diabetes mellitus (diabetic cardiomyopathy, DCM group) with sham operation, (IIb) type 2 diabetes mellitus with myocardial infarction surgery (diabetes with MI, DMI group), and (IIc) type 2 diabetes mellitus with myocardial infarction supplemented with melatonin (DMI supplemented with melatonin, DMI-MEL group). Melatonin (Sigma, St. Louis, MO, USA) was dissolved in absolute ethanol and then diluted in tap water (at a dose of 50 mg/kg body weight/day) [29]. Mice were given melatonin daily immediately following the myocardial infarction procedure till the end of the study. All mice in these three groups were fed high-fat diet for 2 weeks after induction of diabetes prior to the MI or sham procedure. Then, mice were continuously fed with high-fat diet for 4 weeks before sacrifice after the MI surgery or sham operation.

### 2.2. Determination of Blood Glucose Levels

Fasting plasma glucose levels were measured with a blood glucometer (Accu-Chek® Active, Roche Diagnostics, Mannheim, Germany) according to the manufacturer's instruction. Mouse blood was obtained from the tail vein 3 days after STZ injection. According to the previous study [30], mice with FPG > 11.1 mmol/L were confirmed for type 2 diabetes mellitus.

### 2.3. Myocardial Infarction Model

For the myocardial infarction model, the procedure was followed as previously described [31, 32]. In brief, male mice were anesthetized with 1.5% isoflurane by an isoflurane delivery system in the surgical plane. The whole procedure was performed in aseptic conditions, and mice were placed on a heating pad to maintain the body temperature. After a small cut on the left chest, a tiny hole was made in the fourth intercostal space. Then, the heart was squeezed out of the thoracic cavity, and the left coronary artery was ligated using a 6-0 silk suture. The heart was placed back into the intrathoracic space once the ligation was completed. The exposure time for hearts being out of the thoracic cavity was between 30 sec and 1 min. Air was evacuated out of the thoracic cavity prior to the stature of muscles and the skin. The recovery process was under 37°C in an incubator until mice were fully ambulatory. A similar procedure was repeated for the sham-operated group without ligation of the left coronary artery.

### 2.4. Echocardiography

Mice were anesthetized with 1-1.5% isoflurane in oxygen for two-dimensional and motion- (M-) mode echocardiographic measurements at 1 week and 4 weeks, respectively, after surgery, which was performed using the Vevo 2100 high-resolution in vivo imaging system (Visual Sonics, Toronto, ON, Canada). The procedure to evaluate cardiac function was executed blindly. Left ventricular ejection fraction (LVEF) and left ventricular fractional shortening (LVFS) were measured, which were carried out from three consecutive cardiac cycles. Data was calculated on an accompanying workstation with VevoStrain software [33]. Following completion of echocardiography, mice were sacrificed before collection of hearts for further analysis.

### 2.5. Histopathology

Mice were anesthetized and sacrificed. Hearts were obtained and rinsed with an ice-cold PBS and were immediately fixed in 4% polyformaldehyde for 3 days. After that, tissues were immersed in ethanol solutions with various concentrations from 70% to absolute ethanol for dehydration. Then, ethanol in the tissue was displaced by xylene. Then, cardiac tissue was embedded in paraffin and cut in 3 *μ*m sections for histopathological examination. To assess morphological changes, sections were stained with hematoxylin and eosin (H&E). Similarly, sections were stained with Masson's trichrome staining and imaged under the light microscope to evaluate the degree of myocardial interstitial fibrosis. For further analysis, 5 random fields at 200x magnification were chosen for cardiac fibrosis quantification using Image-Pro Plus software. Results were presented using the blue-stained area normalized to the total area excluding endocardial and epicardial regions or perivascular fibrosis [33].

### 2.6. Cell Viability Determination

The Cell Count Kit-8 (CCK-8, Dojindo, Japan) assay was used to determine the effect of melatonin (Tiancheng Biotechnology, Shanghai, China) on H9c2 cell viability and was performed according to the manufacturer's instruction. In brief, H9c2 cells were seeded into 96-well culture plates and were cultured at a density of 4 × 10^3^ cells/well in 200 *μ*L complete medium. Melatonin was dissolved in absolute ethanol at various levels (50 *μ*M, 100 *μ*M, 200 *μ*M, and 300 *μ*M). Vehicle control cells were cultured in DMEM supplemented with 0.1% ethanol. Each treatment group consisted of 5 parallel wells. Then, the medium was replaced after drug treatment, and 10 *μ*L of CCK-8 solution was added to each well and incubated for 2 hr at 37°C. Subsequently, optical density (OD) was measured at a wavelength of 450 nm using a SpectraMax M5 plate reader (Molecular Devices, Sunnyvale, California, USA). Cell viability was calculated as the percentage of the absorbance of the melatonin treated/vehicle control × 100%.

### 2.7. Cell Culture and Treatment

H9c2 cells were cultured in Dulbecco's modified Eagle's medium (DMEM; HyClone, Logan, UT, USA) supplemented with 10% fetal bovine serum (Gibco, Carlsbad, CA, USA) with 100 U/mL streptomycin and 100 U/mL penicillin in an incubator at 95% humidity (37°C with 5% CO_2_). Melatonin (Sigma-Aldrich, Merck Millipore, Darmstadt, Germany) was dissolved in ethanol first and was then diluted in DMEM (5 mM glucose). H9c2 cells were divided into five groups. Cells were cultured in normal DMEM including two groups (groups 1 and 2). In group 1 (control group, CON), cells were cultured in normal DMEM with 0.1% ethanol. In group 2 (ANI), cells were supplemented with 5 *μ*M anisomycin for 2 hr followed by replacement of normal DMEM and were cultured for 14 hr. To examine the effect in diabetic condition, we mimicked the pathological condition with high glucose and high fat (HG/HF, 33 mM glucose and saturated FFA palmitate (16 : 0, 500 *μ*M)) [34]. In group 3 (HG/HF-hypoxia group), cells were cultured in HG/HF medium for 12 hr followed by hypoxia (5% CO_2_, 95% N_2_ humidified atmosphere, yielding less than 1% O_2_ concentrations) for 2 hr. In group 4 (HG/HF-hypoxia-MEL group), cells were cultured in HG/HF medium for 12 hr followed by hypoxia for 2 hr with the addition of melatonin (100 *μ*M) at the second hour. In group 5 (HG/HF-hypoxia-MEL-ANI group), cells were cultured in HG/HF medium under hypoxia and were supplemented with 5 *μ*M anisomycin for 2 hr prior to incubation in HG/HF medium. Following replacement with the HG/HF medium, cells were cultured for 12 hr and were subjected to hypoxic condition for 2 hr along with 100 *μ*M melatonin. Each group consisted of 5 replicates.

### 2.8. TUNEL Staining

The degree of myocardial and cellular apoptosis was measured by TUNEL analysis using an *in situ* cell death detection kit (Roche Molecular Biochemicals, Mannheim, Germany) following the manufacturer's instructions. Nuclei were visualized by DAPI staining. The samples were examined under an Olympus Fluoview FV100 microscope (Olympus, Tokyo, Japan), and the results are presented as an apoptotic index (×100%).

### 2.9. Assessment of Mechanical Properties of Adult Cardiomyocytes

Mice were sacrificed and the hearts were harvested and digested by Liberase Blendzyme (Roche Molecular Biochemicals, Indianapolis, IN, USA). The collected cardiomyocytes were divided into five groups. The cells in the high-glucose/high-fat-hypoxia (HG/HF-hypoxia) group were treated with 33 mM glucose and 500 *μ*M palmitate under hypoxia for 2 hr. To evaluate the role of melatonin, cells in the melatonin (HG/HF-hypoxia-MEL) group were pretreated with 100 *μ*M melatonin for 2 hr before high-glucose/high-fat and hypoxia treatment. The JNK activator anisomycin was used to evaluate the role of JNK in the mechanical properties of adult cardiomyocytes (HG/HF-hypoxia-MEL-ANI). Anisomycin (2 *μ*M) was added to cardiomyocytes for 30 min prior to melatonin treatment.

Following respective drug treatment, only rod-shape cardiomyocytes were used for mechanical assessment. Cardiomyocytes were visualized using an inverted microscope (IX-70, Olympus, Tokyo, Japan), and mechanical properties of cardiomyocytes were assessed by using a SoftEdge MyoCam system (IonOptix Corporation, Milton, MA, USA). The indices of peak shortening, maximal velocity of shortening and relengthening (±dL/dt), time-to-peak shortening (TPS), and time-to-90% relengthening (TR_90_) were measured to evaluate the mechanical properties of adult cardiomyocytes [33].

### 2.10. Western Blot

Lysis buffer was prepared with protease inhibitors and phosphatase inhibitor cocktail (Roche, Shanghai, China) after cardiac tissues and H9c2 cardiomyocytes were collected. After homogenizing the samples, the lysates were centrifuged at 12,000 rpm for 15 min at 4°C. Protein concentration was quantified by a BCA protein assay kit, and then, the protein sample was separated by SDS-PAGE. Proteins were transferred to a polyvinylidene difluoride (PVDF) membrane (Merck Millipore, Darmstadt, Germany). After blocking the PVDF membrane with 5% nonfat milk for 2 hr at room temperature (RT), the membranes were incubated with primary antibodies against p-JNK, JNK, p53, Bcl-2, Bax, caspase-3, and GAPDH (Cell Signaling Technology, Beverly, MA, USA) at 4°C overnight. After washing with TBS containing 0.1% Tween 20 (TBST) five times for 10 min each, the membranes were incubated with secondary antibodies in TBST buffer for 2 hours at RT and washed with TBST as previously described. The bands were visualized by adding the chemiluminescent HRP substrate (Merck Millipore, MA, USA) to the membrane, and the signals were detected by a ChemiDoc system (Bio-Rad, Richmond, USA) and quantified by using Image Lab software (Bio-Rad, Richmond, USA) [33].

### 2.11. Statistical Analysis

All data were presented as means ± standard error of the mean (SEM). Differences were compared using a one-way ANOVA followed by the Bonferroni correction for *post hoc* analysis. A *p* value < 0.05 was considered to be statistically significant. All statistical analyses were performed using GraphPad Prism software (GraphPad Software, San Diego, CA, USA).

## 3. Results

### 3.1. Echocardiogram of Diabetic Mice following MI with or without Melatonin Treatment

**Figure 1:**
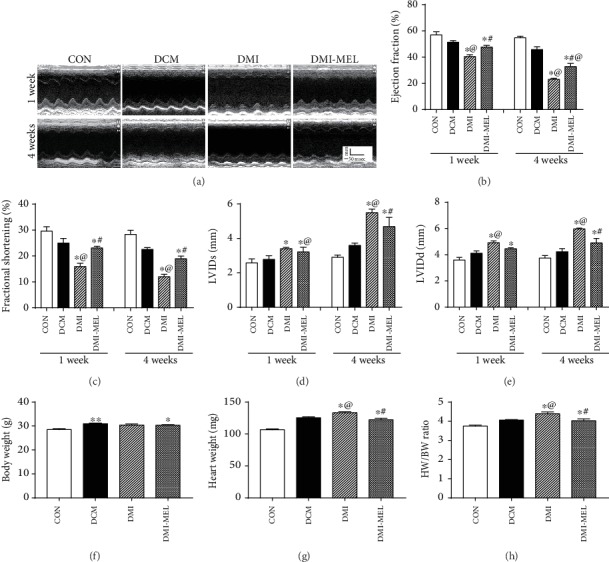
Effect of melatonin treatment on cardiac contractile function in post-MI diabetic mice. (a) Representative M-mode echocardiographic images from four respective mouse groups. Cardiac function was assessed using echocardiography at the first and fourth weeks post-MI. (b) Ejection fraction. (c) Fractional shortening. (d) LVIDs: left ventricular internal dimension during systole (end-systolic diameter). (e) LVIDd: left ventricular internal dimension during diastole (end-diastolic diameter). (f) Body weight. (g) Heart weight. (h) Heart weight-to-body weight (HW/BW) ratio. CON: normal control; DCM: diabetic cardiomyopathy; DMI: diabetes mellitus with MI; DMI-MEL: diabetes mellitus with MI treated with melatonin. Mean ± SEM, *n* = 4-6. ^∗^*p* < 0.05 vs. CON group, ^#^*p* < 0.05 vs. DMI group, and ^@^*p* < 0.05 vs. DCM group.

Cardiac function and geometry were evaluated in post-MI diabetic mice with ejection fraction (EF) and fractional shortening (FS) assessed using echocardiography at the first and fourth weeks. Results shown in Figures 1(a)–1(e) demonstrated the minimal effects of diabetes on cardiac geometry and function compared to the control group although post-MI imposed overt functional and geometric alterations in the diabetic state (manifested by reduced ejection fraction, fractional shortening, and enlarged LVIDs and LVIDd at both 1- and 4-week time points) compared to mice in the DCM group, the effect of which was significantly attenuated or negated by melatonin treatment (except for the elevated LV geometry at 1-week post-MI). These data indicated that melatonin treatment improved cardiac geometry and contractile function at various time points (with a more pronounced effect with longer duration) post-MI in diabetic condition. In addition, results in Figures 1(f)–1(h) depicted that body weight was overtly elevated in the diabetic group, the effect of which was spared by the MI procedure and melatonin treatment. Although diabetes failed to significantly alter heart weight or heart size (heart-to-body weight ratio), the MI procedure overly elevated heart weight and heart size in the diabetic model, the effect of which was significantly attenuated by melatonin treatment in the diabetic state.

### 3.2. Melatonin Reduced Cardiac Fibrosis in the Border Zone of the Infarcted Area Post-MI in Diabetic Mice

**Figure 2:**
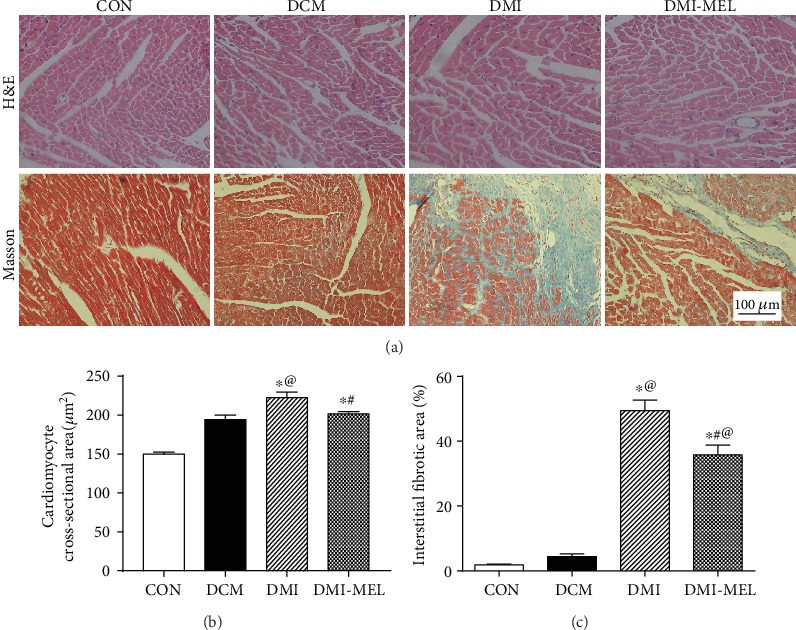
Effect of melatonin on the cardiomyocyte cross-sectional area and cardiac fibrosis with high-fat diet and/or myocardial infarction. (a) H&E staining and Masson's trichrome staining. The upper panel exhibits representative images of H&E staining of cardiac tissues. The lower panel depicts representative images of cardiac interstitial fibrosis with Masson's trichrome staining. (b) Pooled data of the cardiomyocyte cross-sectional area (*n* = 20-25 cells per group). (c) Pooled data of the interstitial fibrotic area of myocardial tissues. CON: normal control; DCM: diabetic cardiomyopathy; DMI: diabetes mellitus with MI; DMI-MEL: diabetes mellitus with MI treated with melatonin. Mean ± SEM, *n* = 3-4 per group. ^∗^*p* < 0.05 vs. CON group, ^#^*p* < 0.05 vs. DMI group, and ^@^*p* < 0.05 vs. DCM group. Scale bar = 100 *μ*m.

To determine whether melatonin administration alleviated cardiac fibrosis post-MI in diabetic mice, cardiac tissue was examined using H&E staining and Masson's trichrome staining. Results shown in Figures 2(a)–2(c) suggested that diabetes alone failed to significantly affect the cross-sectional area of cardiomyocytes compared with the control group. However, the cross-sectional area of cardiomyocytes was overtly elevated following MI in diabetes compared to the diabetic-alone group, the effect of which was alleviated by melatonin treatment in the diabetic state. Besides, the cardiac interstitial fibrotic area in the infarct border zone evaluated by Masson's trichrome staining depicted a significant rise in the post-MI diabetes group compared to the diabetic-alone group although diabetes alone did not significantly increase myocardial fibrosis compared with the control group as shown in Figures 2(a)–2(c). Compared to the DMI group, melatonin administration markedly attenuated cardiac interstitial fibrosis in the diabetic state. These results collectively demonstrated that melatonin mitigated MI-induced rises in the cardiomyocyte cross-sectional area and cardiac interstitial fibrotic area in diabetes.

### 3.3. Melatonin Treatment Preserved Heart Function by Reducing Apoptosis Post-MI in Diabetic Mice

**Figure 3:**
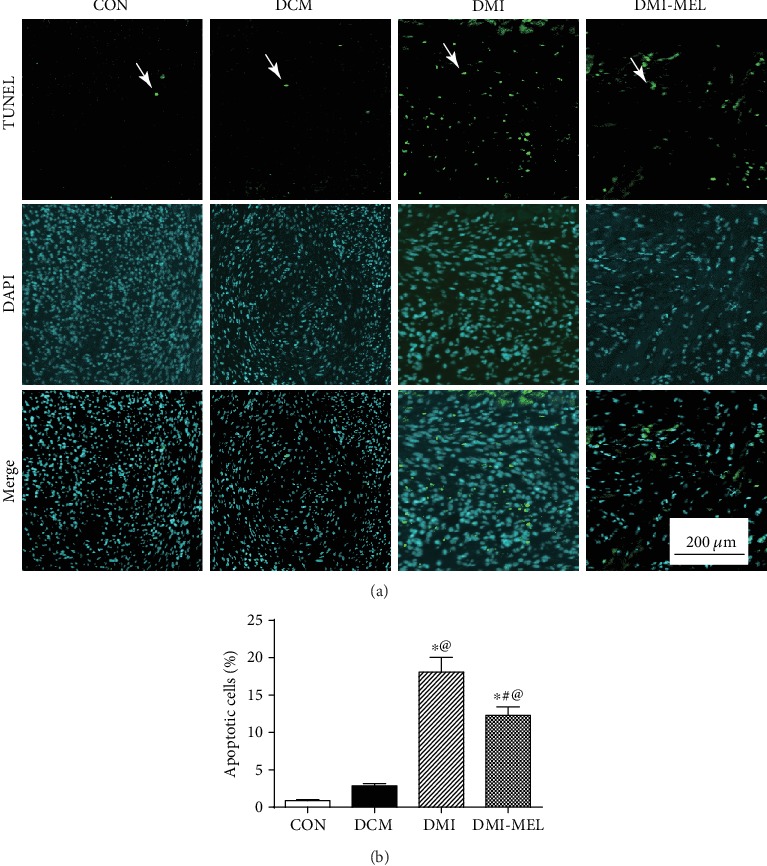
Effect of melatonin treatment on myocardial TUNEL apoptosis in post-MI diabetic mice. (a) Representative images of TUNEL staining 4 weeks post-MI in diabetic mice. Representative TUNEL staining images are displayed in the upper panel while the images with DAPI staining are displayed in the middle panel demonstrating nuclei of cells. The lower panel exhibits the overlay images to illustrate apoptotic cells. (b) Pooled data displaying the percentage of apoptotic cells. Mean ± SEM, *n* = 3-5 per group. ^∗^*p* < 0.05 vs. CON group, ^#^*p* < 0.05 vs. DMI group, and ^@^*p* < 0.05 vs. DCM group. Scale bar = 200 *μ*m.

Myocardial apoptosis was assessed using TUNEL staining to demonstrate the protective effects of melatonin in post-MI diabetic mice. Results in Figures 3(a) and 3(b) showed that MI greatly enhanced myocardial apoptosis compared with the diabetic group although diabetes alone did not significantly increase cellular apoptosis compared with the control group. However, apoptotic cells were significantly decreased following melatonin treatment in the diabetic state, demonstrating an inhibitory effect of melatonin on cardiomyocyte apoptosis post-MI in diabetic hearts *in vivo*.

### 3.4. Melatonin Reduced Cardiac Apoptosis via Inhibiting the JNK Pathway Post-MI in Diabetic Mice

**Figure 4:**
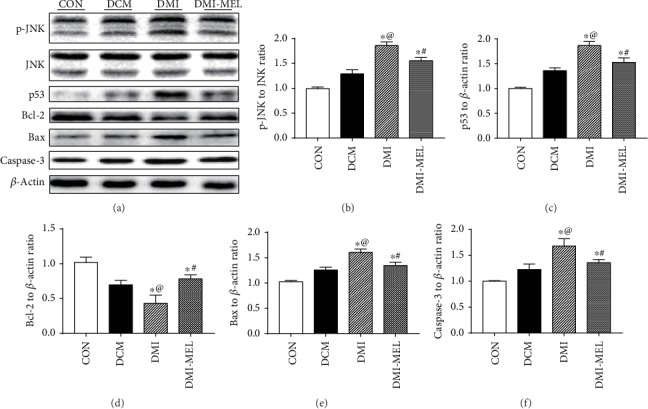
Effect of melatonin on JNK and apoptotic-related protein expressions with or without melatonin treatment in diabetic MI hearts. (a) Representative images of western blot 4 weeks post-MI in diabetic mice. (b) p-JNK to total JNK ratio. (c) p53 level. (d) Bcl-2 level. (e) Bax level. (f) Caspase-3 level. Mean ± SEM, *n* = 3-5 per group. ^∗^*p* < 0.05 vs. CON group, ^#^*p* < 0.05 vs. DMI group, and ^@^*p* < 0.05 vs. DCM group.

Levels of JNK and apoptotic proteins were further examined in post-MI diabetic mice with or without melatonin treatment. Results shown in Figures 4(a)–4(f) revealed that the post-MI procedure markedly promoted JNK phosphorylation in the diabetic group compared with the diabetic-only group although diabetes alone failed to notably alter levels of JNK phosphorylation compared with the control group. Furthermore, rises in p53, caspase-3, and Bax levels along with a drop in Bcl-2 level were observed in the diabetic group following MI compared with the diabetic-only or control group. However, these changes were reversed by melatonin treatment in the diabetic state. These data collectively demonstrated that melatonin could alleviate cardiac injury in post-MI diabetic mice through inhibition of the JNK/p53-mediated apoptotic pathway.

### 3.5. Melatonin Decreased JNK Phosphorylation to Inhibit Cardiomyocyte Apoptosis In Vitro under Hypoxia with High-Glucose/High-Fat Insult

**Figure 5:**
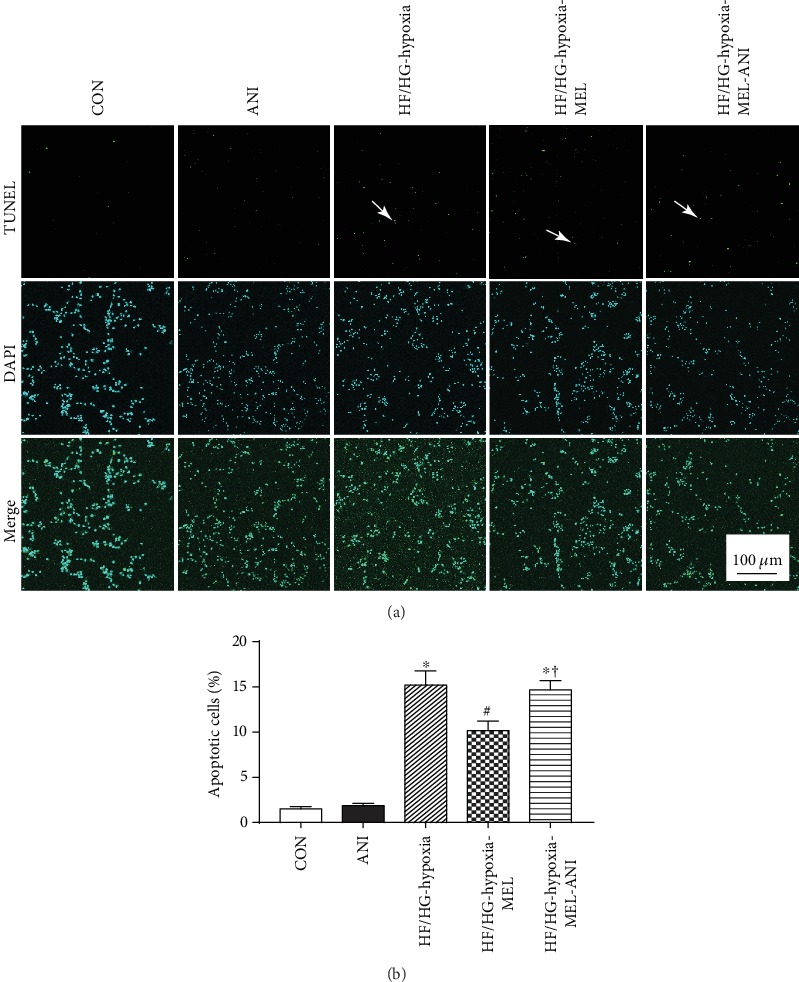
The effect of melatonin treatment on cellular apoptosis in H9c2 myocytes challenged by high glucose/high fat under hypoxia. (a) Representative images of TUNEL staining of H9c2 cells with or without melatonin and anisomycin treatment. The images with TUNEL staining are displayed in the upper panel. Images with DAPI staining are displayed in the middle panel depicting nuclei of cells. Lower panel indicates the overlay images to illustrate the apoptotic cells. (b) Pooled data depicting the percentage of apoptotic cells. Mean ± SEM, *n* = 3 per group. ^∗^*p* < 0.05 vs. CON group, ^#^*p* < 0.05 vs. HF/HG-hypoxia group, ^†^*p* < 0.05 vs. HF/HG-hypoxia-MEL group. Scale bar = 100 *μ*m.

To verify the role of the JNK pathway in the pathological progress of diabetic post-MI hearts, we further tested the effects of JNK activation on cellular apoptosis *in vitro* in H9c2 myocytes under hypoxia combined with high-glucose/high-fat treatment. Our results in Figures 5(a) and 5(b) showed that high-glucose/high-fat insult combined with hypoxia significantly triggered cellular apoptosis compared with the control group. However, melatonin treatment greatly reduced cellular apoptosis compared with the HF/HG-hypoxia group. Interestingly, the protective role of melatonin was negated in the presence of the JNK activator anisomycin in high-glucose/high-fat treatment under hypoxia. These data consolidated that melatonin overtly reduced cell apoptosis at least in part via JNK inhibition in the face of high-glucose/high-fat and hypoxia insult.

### 3.6. Melatonin Protects Cardiomyocytes from Apoptosis Induced by Hypoxia and High-Glucose/High-Fat Treatment via the JNK-Mediated Apoptosis Pathway

**Figure 6:**
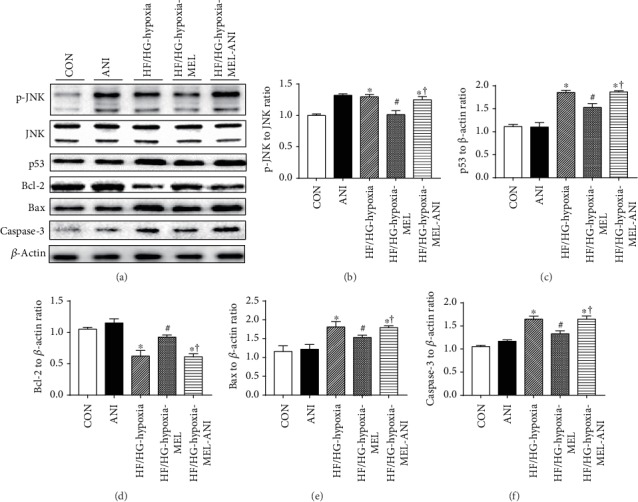
Effect of melatonin and the JNK activator anisomycin on protein levels of apoptotic-related proteins in H9c2 cells challenged by high glucose/high fat under hypoxia. (a) Representative images of western blot of H9c2 cells with or without melatonin and anisomycin treatment. (b) p-JNK to total JNK ratio. (c) p53 level. (d) Bcl-2 level. (e) Bax level. (f) Caspase-3 level. Mean ± SEM, *n* = 3. ^∗^*p* < 0.05 vs. CON group, ^#^*p* < 0.05 vs. high-glucose/high-fat treatment under hypoxia, and ^†^*p* < 0.05 vs. HF/HG-hypoxia-MEL group.

We further examined levels of JNK and related apoptotic proteins in H9c2 cells treated with the JNK activator anisomycin under hypoxia in conjunction with high-glucose/high-fat insult. In line with our results of TUNEL staining, results in Figures 6(a)–6(f) showed that high glucose/high fat in combination with hypoxia significantly increased JNK phosphorylation and levels of p53, Bax, and caspase-3 and reduced the expression of Bcl-2, the effects of which were reversed by the addition of melatonin. However, anisomycin treatment partially attenuated the protective effects of melatonin and reversed the changes of protein levels, confirming an obligatory role of the JNK pathway in melatonin-produced protection against apoptosis in the face of high-glucose/high-fat and hypoxia insult.

### 3.7. Melatonin Improved Contractile Properties of Adult Cardiomyocytes Insulted by Hypoxia and High-Glucose/High-Fat Treatment

**Figure 7:**
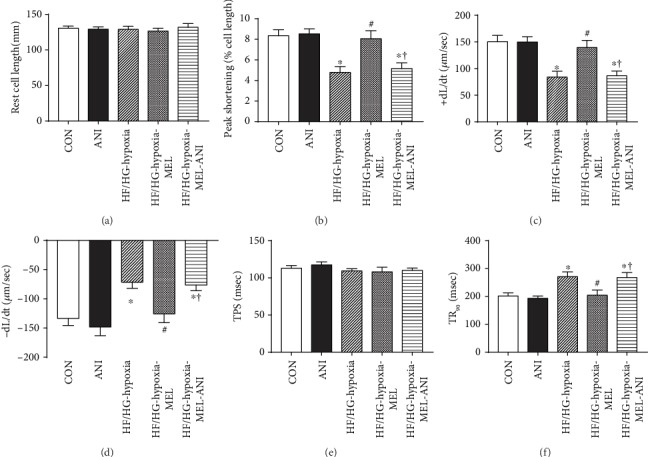
Effect of melatonin and the JNK activator anisomycin on contractile function of isolated cardiomyocytes. (a) Resting cell length. (b) Peak shortening (PS, normalized to resting cell length). (c) Maximal velocity of shortening (+dL/dt). (d) Maximal velocity of relengthening (−dL/dt). (e) Time-to-peak shortening (TPS). (f) Time-to-90% relengthening (TR_90_). Mean ± SEM, *n* = 39 cells per group. ^∗^*p* < 0.05 vs. CON group, ^#^*p* < 0.05 vs. HF/HG-hypoxia group, and ^†^*p* < 0.05 vs. HF/HG-hypoxia-MEL group.

To clarify the role of the JNK pathway in melatonin-mediated protection of cardiomyocytes, isolated adult murine cardiomyocytes were treated with high glucose/high fat and hypoxia in the presence or the absence of melatonin and JNK activator anisomycin. Results shown in Figures 7(a)–7(f) displayed that high-glucose/high-fat and hypoxia treatment markedly decreased peak shortening, maximal velocity of shortening/relengthening, and prolonged relengthening time without affecting resting cell length and time-to-peak shortening. Furthermore, melatonin overtly improved cardiomyocyte contractile properties under HF/HG-hypoxia challenge. Although anisomycin alone did not exhibit any effect on these mechanical indices, it nullified melatonin-offered protection against HF/HG-hypoxia-induced cardiomyocyte anomalies. Our data revealed the involvement of the JNK pathway in melatonin-offered protection against HG/HF-hypoxia-elicited contractile anomalies.

## 4. Discussion

The salient findings from our present study revealed that administration of melatonin ameliorated post-MI-induced cardiac dysfunction and interstitial fibrosis in the diabetic state. Post-MI diabetes elicited decreased ejection performance of the left ventricle, increased LV dilation, worsened cardiac fibrosis of the border zone, and exacerbated apoptosis in the heart. Further scrutiny depicted that melatonin-offered benefits were accompanied by alterations in the levels of phosphorylated JNK, p53, Bcl-2, Bax, and caspase-3. These findings along with the *in vitro* findings convincingly indicated that the JNK activator anisomycin nullified melatonin-induced cardioprotection against high-glucose/high-fat-hypoxia-induced cardiac biochemical and functional deficits, validating the role for the JNK pathway in melatonin-offered benefit against post-MI in diabetes.

Over the past decades, the prevalence of type 2 diabetes is dramatically increasing globally in both developed and developing countries [1, 35]. In Asia, type 2 diabetes occurs at a much younger age with lower degree of obesity and has a higher mortality rate compared with that in other regions [36–38]. While diabetes is considered a substantial risk of cardiovascular disease [38], evidence has shown that the risk of developing cardiovascular diseases in patients with type 2 diabetes was twice higher than the risk in those without type 2 diabetes [39]. Despite the fact that ample studies clarified the potential molecular mechanisms of MI, the unique features of the pathogenesis of MI in diabetic hearts remain elusive. Findings from earlier studies revealed that diabetes may downregulate the expression of Sirt3 and disrupt the Ang-1/Tie-2 signaling cascade to enhance myocardial infarction injury via regulation of myocardial vascular maturation and angiogenesis [40, 41]. Importantly, enhanced apoptosis of cardiomyocytes contributed to the exacerbation of cardiac function and fibrosis in diabetic MI compared to nondiabetic MI settings in rats [42]. Involvement of JNK stress signaling has been considered over the past decades. As a well-characterized member of the MAPK superfamily, JNK participates in the pathogenesis of cardiovascular diseases such as myocardial infarction, heart failure, myocardial ischemia-reperfusion (MI/R) injury, diabetic cardiomyopathy, dilated cardiomyopathy, and cardiac hypertrophy [43–47]. In particular, overactivation of JNK *in vivo* can cause restrictive cardiomyopathy and cardiac fibrosis, as well as leading to conduction defects and heart failure [48–50]. JNK is also considered an essential modulator for mitochondrial homeostasis and apoptosis in the onset and progression of heart failure [47, 51, 52]. Activation of JNK/p38 cascades has been demonstrated to aggravate the development of MI/R injury [53]. However, the roles of JNK in diabetic MI hearts were not fully examined with recent findings favoring the role of JNKs in I/R injury of the heart in diabetes mellitus [54]. Nonetheless, clinical application of JNK inhibitors for target therapy has been dismal due to family subtype specificity and toxicity [54]. In our hands, the MI procedure greatly increased phosphorylation of JNK in diabetes compared with the diabetes-alone group (which exerts no significant effect on JNK phosphorylation). Our data also revealed prominent apoptosis in conjunction with JNK activation in the diabetic MI group. Levels of antiapoptotic protein Bcl-2 were decreased while those of p53, Bax, and caspase-3 were elevated in post-MI diabetic hearts compared to control or diabetic-alone group. Our TUNEL staining result also showed that the apoptosis is markedly increased in diabetic post-MI hearts. We further demonstrated the enhanced cell apoptosis through JNK activation in H9c2 cells with HG/HF and hypoxia treatment. These data together illustrated that apoptosis activated by the JNK pathway is important in the pathogenesis of MI of diabetic hearts.

**Figure 8:**
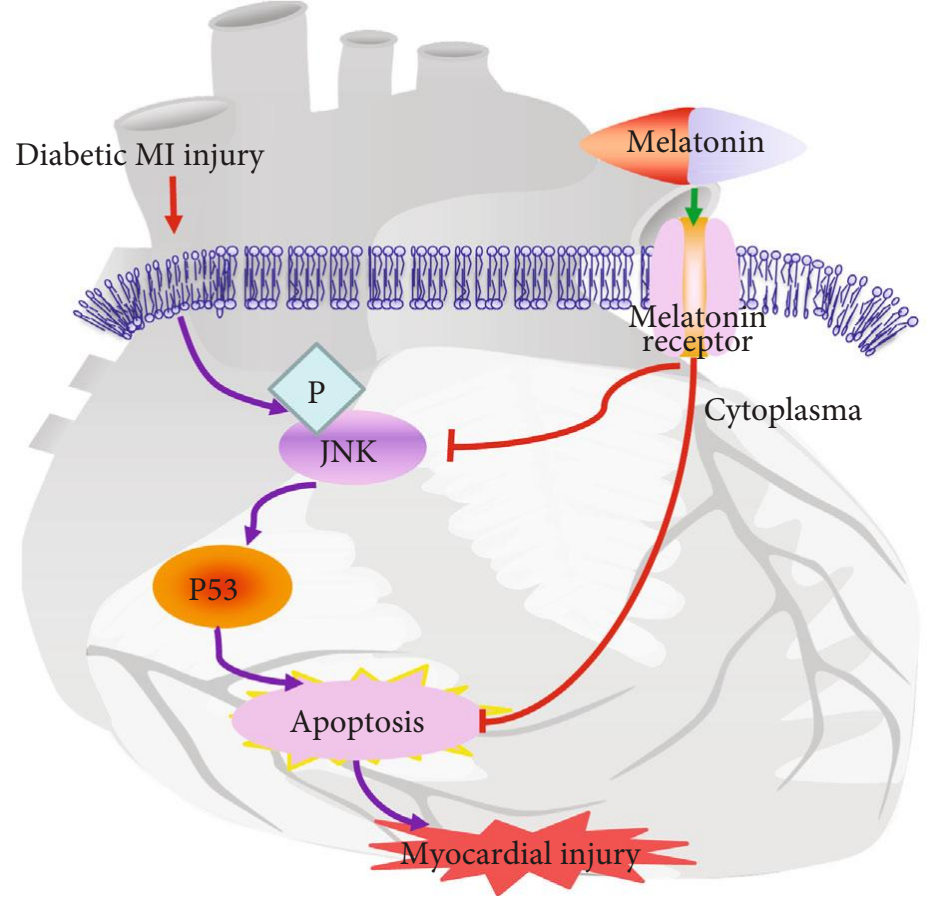
Schematic diagram depicting the proposed signaling pathways involving JNK, p53, and apoptosis behind the melatonin-offered protective effect against post-MI injury in diabetes.

As a hormone secreted by the pineal gland, poor secretion of melatonin was associated with a high risk of type 2 diabetes, implying the potential benefits of melatonin in metabolic diseases [55]. Besides, melatonin significantly alleviated metabolic disorder and oxidative stress in the diabetic state [56, 57]. Furthermore, 6melatonin was shown to protect diabetic hearts via an apoptosis- and Mst1/Sirt3-dependent mechanism [58, 59]. In our hands, blood glucose level of the DMI-MEL group was comparable between diabetic and diabetic MI groups (data not shown), suggesting a lack of effect for melatonin on blood glucose in the diabetic state. It was noticed that the heart weight in the DMI-MEL group dropped although body weight in the DMI-MEL group did not significantly change compared to that in the DMI group. Although LVEF and LVFS values were overtly lowered in the diabetic MI group compared with the diabetic group, melatonin treatment restored LVEF and LVFS values in the diabetic MI group. Compared with the diabetic MI group, melatonin significantly reduced myocardial fibrosis of the infarct border zone and cross-sectional area of cardiomyocytes. These results favored that administration of melatonin significantly decreased phosphorylation of JNK and apoptosis (manifested by apoptotic proteins such as Bax and caspase-3 and TUNEL staining), in line with the previous study [60]. We further validated the obligatory role of JNK in melatonin-offered cardioprotection using anisomycin to nullify the protective effect of melatonin. Although high glucose/high fat in combination with hypoxia greatly impaired the mechanical properties of cardiomyocytes, melatonin overtly improved the contractile function of murine cardiomyocytes, the effects of which were nullified by anisomycin. These data collectively demonstrated that melatonin treatment effectively protected the hearts from MI injury in diabetic mice possibly through a JNK-mediated mechanism as depicted in Figure 8.

In summary, findings from our present study demonstrate that JNK signaling is turned on in post-MI diabetic hearts. Administration of melatonin is capable of improving post-MI diabetes-induced cardiac dysfunction and myocardial fibrosis of the infarct border zone in the diabetic state, possibly through a mechanism associated with JNK-mediated apoptosis. Our data should shed some lights on a better exploration of novel therapeutic targets and development of the potential clinical use of melatonin on ischemic cardiovascular diseases with diabetes.

## Data Availability

The data used to support the findings of this study are available from the corresponding authors upon request.
